# Relationship of Exercise Volume with Change in Depression and Its Association with Self-Efficacy to Control Emotional Eating in Severely Obese Women

**DOI:** 10.4061/2011/514271

**Published:** 2011-03-14

**Authors:** James J. Annesi, Linda L. Vaughn

**Affiliations:** Department of Wellness, YMCA of Metropolitan Atlanta, 100 Edgewood Avenue NE, Suite 1100 Atlanta, GA 30303, USA

## Abstract

*Introduction*. Exercise may improve one's perceived ability to control overeating related to negative emotions through psychological pathways such as reduced depression; however, the volume required is unclear. *Methods*. Severely obese women (*N* = 88) participated in a 24-week exercise and nutrition treatment incorporating self-regulatory skills training, and were assessed on depression, self-efficacy, self-regulatory skills usage, weight, and waist circumference, at baseline and treatment end. *Results*. Subjects completing low-moderate (40–149.9 minutes/week) and public health (≥150 minutes/week) volumes of exercise had significant and similar reductions in depression scores. No significant changes were found for those completing <40 minutes/week. For all subjects aggregated, depression change was significantly related to change in self-efficacy to control emotional eating; however, this relationship was completely mediated by changes in self-regulatory skill usage. When changes in depression, self-efficacy, and self-regulatory skills usage were entered into multiple regression equations as predictors, only self-regulatory skill changes explained significant unique portions of the overall variance in weight and weight circumference change. *Discussion*. Exercise of less than half the public health recommendation was associated with depression improvement, with no dose-response effect. Changes in depression, self-efficacy, and self-regulation may be salient variables to account for in behavioral weight-loss treatment research.

## 1. Introduction

A better understanding of the behavioral dynamics of overeating is required for more effective and efficient weight management intervention. Emotional eating, for example, is now a well-documented phenomenon [1] and may be most prevalent in females [2]. It has, however, been difficult to directly measure and account for in weight-loss research [3]. Perceived ability/inability to control overeating when negative moods are present may be more effectively measured and has been associated with weight loss/gain [4, 5]. 

Physical activity is the best predictor of maintained weight loss [6, 7]. However, research indicates that only a very small portion of weight lost is directly attributable to associated caloric expenditures [8], which tend to be especially low in obese and deconditioned individuals initiating exercise [9, 10]. Exercise is a type of physical activity that is planned and structured. It is proposed that exercise-induced improvements in negative psychological states, such as depression, are a major factor in an association between physical activity, controlled eating, and weight loss [8, 11]. It is possible that exercise-induced reductions in depression are related to increased feelings of ability to control emotional eating (self-efficacy for controlling emotional eating). 

There remains a debate on the etiology of exercise-induced changes in depression [12, 13]. Biochemical theories suggest that associated changes in endorphin, serotonin, and norepinephrine levels induce improvements; thus, there would be a dose-response effect (i.e., more exercise—more reduction in depression). Conversely, behavioral theories suggest that simply participating in a program of physical activity fosters self-efficacy to manage one's self through barriers that lead to a generalized sense of well-being and improved mood. Studies have supported both positions. For example, Dunn and colleagues found that a minimum of 5 days/week of moderate cardiovascular exercise (termed “public health dose”) over 12 weeks was required for a significant improvement in depression, with 3 days/week or less demonstrating no significant change [14]; while Annesi found that participation for as little as 2 or 3 days/week over 10 weeks, with 20–25-minute or 15-minute bouts at a moderate intensity, respectively, was associated with significant reductions in depression scores [15], with a dose-response relationship not present. Because self-report data suggests that only 19% and 27% of overweight and obese women, respectively, in the U.S. maintain the public health dosage of at least 150 minutes/week (e.g., 5 days/week × 30 minutes per day of moderate physical activity) [16], yet 64% are either overweight or obese [17], this volume may present an adherence problem if included as a treatment component for weight loss.

Exercise treatments with behavioral components may seek to improve self-regulatory and self-management skills that might carry over to confidence in controlled eating [18], possibly through effectively dealing with negative emotions [8]. Thus, such self-regulation may serve to mediate a relationship between depression and self-efficacy to control eating when negative emotions are present. Relative effects of changes in depression, self-efficacy, and self-regulation on weight and body composition, however, remain unknown. 

Thus, the following research questions required further investigation to improve weight-loss research, theory, and treatment.

1. What is the volume of exercise required to induce a significant reduction in measured depression?
2. Regardless of exercise volumes completed, are depression changes significantly associated with self-efficacy to control negative emotion-related overeating, and, if so, does self-regulatory skill usage mediate this relationship?
3. Regardless of exercise volumes completed, does change in depression alone, and/or changes in depression along with self-efficacy and self-regulation change, predict weight and waist circumference change?

Because of either a lack of related research or inconsistent findings, each of these concerns is given as a research question, without hypotheses. Hopefully, the mechanisms by which depression may be associated with weight change, and how exercise may best be used, could be clarified. An exercise treatment based on social cognitive and self-efficacy theory was chosen because improvements in the psychological variables of interest would be focused upon. Severely obese women were recruited as subjects because they may be most affected by emotional eating when attempting weight loss.

## 2. Methods

### 2.1. Subjects

Women responded to advertisements in local newspapers for participation in research incorporating physical activity and nutrition instruction for weight loss. Requirements were age ≥21 years, body mass index (BMI) of 35–50 kg/m^2^ (class 2 and 3 obesity), and no regular exercise reported (<20 minutes/week) in the previous year. Present or planned pregnancy and use of medication for weight loss or a psychiatric condition were causes for exclusion. A written statement of adequate physical health to participate was required from a physician. Institutional review board approval and written consent from subjects was obtained. The 88 subjects (Mean_age_ = 41.9, SD = 10.1 years; Mean_BMI_ = 42.5, SD = 6.2 kg/m^2^; racial makeup of 54% White, 43% African American, and 3% of other races/ethnicities) were mostly in the lower-middle to middle class.

### 2.2. Measures

The Profile of Mood States Short Form scale of depression [19] has 5, 1-word items (e.g., sad, gloomy) that require responses from 0 (not at all) to 4 (extremely) indicating, “… how you have been feeling during the past week including today.” For women, Chronbach's *α* = .95, and test-retest reliability over 20 days was  .74. Concurrent validity was demonstrated with a variety of accepted measures [19].

The Weight Efficacy Lifestyle Scale subscale of negative emotions assesses self-efficacy to control eating when negative emotions are present through 4 items (e.g., “I can resist eating when I have experienced failure”) [4]. Item responses range from 0 (not confident) to 9 (very confident), with *α* = .87-.88 [4].

Usage of self-regulation skills for appropriate eating was measured using a version of a scale by Saelens and colleagues [20]. Consistent with guidelines, items were based on the content of the self-regulatory skills portion of the treatment. Responses to 10 items ranged from 1 (never) to 5 (often) (e.g., “I keep a record of my eating,” “I say positive things to myself about eating well”). Internal consistency (*α* = .75), test-retest reliability over 2 weeks (.77), and predictive validity were supported [21]. For the present version, *α* = .81, and the test-retest reliability was  .74, in pilot research. 

A recently calibrated digital scale was used to measure weight (kg), and a tape measure was used to measure waist circumference (cm) at the umbilicus.

Changes were calculated by subtracting the baseline score from the score at treatment end (week 24).

### 2.3. Procedure

Participants received access to YMCA wellness centers and were enrolled in a nutrition and exercise treatment based on tenets of social cognitive and self-efficacy theory. The exercise support portion of the treatment consisted of 6 structured, 1-on-1 meetings of 45–60 minutes each, over 24 weeks. It was supported by a previously tested computer program [22]. Instruction in an array of self-regulatory methods (e.g., long- and short-term goal setting with logging of incremental progress, thought-stopping and cognitive restructuring, stimulus control, self-reward, preparing for specific barriers, recovery from lapses) was the primary focus. Cardiovascular exercise plans (e.g., modality, duration, and intensity) were based on each subject's preference and tolerance; however, the volume suggested for health promotion (5 days × 30 minutes/week or 150 minutes/week) [23] was described along with a statement that *any* volume of exercise may have benefit.

The nutrition portion of the treatment consisted of 6 structured, 1-hour group sessions over the initial 12 weeks [24]. Components included (1) understanding macronutrients, (2) using the US Food Guide Pyramid, (3) developing a plan for snacking, and (4) use of self-regulation skills. The self-regulation skills addressed were similar to those in the exercise component, but were focused on managing eating behaviors. 

Subjects self-reported volumes of exercise completed immediately after each bout either at a kiosk at the YMCA facility or elsewhere through the Internet. The electronic method used demonstrated reliability and predictive validity in previous research [8]. Fidelity of treatment protocols was monitored by YMCA wellness administrators under the direction of a study investigator. Assessments were administered in a private area by certified wellness professionals at baseline and at treatment end.

### 2.4. Data Analyses

For the initial analysis, subjects' mean weekly volumes of exercise completed over the course of the investigation were calculated. Subjects were grouped based on their being nonadherent (<40 minutes/week; *n* = 33), or completing low-moderate (40–149.9 minutes/week; *n* = 30) or the public health (≥150 minutes/week; *n* = 25) volumes of moderate-intensity exercise (adjustments were made for the few cases where exercise was of a very low or high intensity over the course of the study). In previous research, Annesi [15] found what is being termed here as the “low-moderate” volume, and Dunn and colleagues [14] found what is being termed the “public health” volume, as the minimum required to induce a significant reduction in depression. A mixed-model repeated measures analysis of variance (ANOVA) was calculated to assess if depression scores changed over the 24-week treatment, and if those changes significantly differed by the 3 groupings of exercise volumes completed. When a significant difference was identified, effect sizes were measured using partial eta squared (*η*_p_^2^) where  .01  =  small,  .06 = moderate,  and  .14 = large effects. 

For subsequent analyses, results from all 88 subjects were aggregated. Changes in depression, self-efficacy to control eating when negative emotions are present (Weight Efficacy Lifestyle Scale—negative emotions subscale), self-regulatory skill use for eating, weight, and waist circumference were next calculated. Use of change scores, rather than scores at a single temporal point, has been suggested to best account for the dynamic nature of the weight-loss process and its predictors [25]. Actual scores, rather than scores adjusted for baseline values, were used to best account for changes associated with a naturally occurring range of initial scores in the present sample type. This is consistent with previous related research when data are normally distributed and free from ceiling and floor effects [25–27]. 

The linear bivariate relationship between changes in depression and self-efficacy to control eating when negative emotions are present was then derived through regression analysis. Mediation analysis was then conducted to determine if changes in self-regulation for eating significantly mediated this relationship. Although methods are available to adapt data with nonnormal distributions for mediation analyses (e.g., bootstrapping) [28], the method proposed by Baron and Kenny [29], utilizing the Sobel test for mediation [30], was appropriate here because a conservative approach for normal distributions was sought. 

Finally, 2 hierarchical multiple regression analyses were calculated. In step 1 of the first equation, change in depression was entered as a predictor of weight change. In step 2, changes in self-efficacy to control eating when negative emotions are present, and self-regulation for eating, were forced into the equation as additional predictors to determine if a significant increase in the explained variance in weight change occurred. In the second multiple regression equation, change in waist circumference replaced weight change as the dependent variable. 

An intention-to-treat design, that retained data from all the original subjects, was used with missing data (17% of all cases) imputed using the last-observation-carried-forward approach, which is common in similar research [27, 31]. Statistical significance was set at *α* = .05 (2-tailed), with the Bonferroni adjustment applied where appropriate. Analyses were conducted using SPSS version 15.0.

## 3. Results

### 3.1. Preliminary Analyses

For all 88 subjects aggregated, significant improvements were found on all measures (Table 1), and change scores in the 3 psychological measures that served as predictors of weight and waist circumference change were approximately normally distributed (<3 SE for skewness; <10 SE for kurtosis) [32].

### 3.2. Changes in Psychological and Physiological Variables

For depression scores, there was no significant difference between the non-adherent, low-moderate exercise volume, or public health exercise volume groups at baseline, *F*(2, 85) = 1.91, *P* = .16. Changes over 24 weeks were significant, *F*(1, 85) = 9.66,  *P* = .003,   *η*_p_^2^ = .102, and significantly differed by group, *F*(2, 85) = 7.76, *P* = .001, *η*_p_^2^ = 0.154. Bonferroni follow-up tests indicated that both the groups of low-moderate and public health volumes of exercise demonstrated more reduction in depression than the non-adherent group, but their changes did not significantly differ from one another.

### 3.3. Relation of Changes in Depression and Self-Efficacy to Control Eating When Negative Emotions Are Present

For all subjects aggregated, the relation between changes in depression and self-efficacy to control eating when negative emotions are present (path c) was significant, *β* = −.30, SE =  .31, *P* = .01. Change in self-regulation for eating was a significant mediator of this relationship (*Z* = 3.81, *P* < .001), and reduced the original relationship to no longer being significant (path c′; *β* = −.02, SE  =  .30, *P* = .87). Thus, complete mediation was observed. Within this mediation analysis, significant relationships were found between both changes in depression and self-regulation for eating (path a; *β* = −.50, SE  =  .22, *P* < .001) and changes in self-regulation for eating and self-efficacy to control eating when negative emotions are present (path b; *β* = .57, SE  =  .13, *P* < .001).

### 3.4. Relations of Change in Depression with Weight and Weight Circumference

The relation of change in depression with weight change was significant (Table 2). Stepping in changes in self-efficacy to control eating when negative emotions are present, and self-regulation for eating, significantly strengthened the amount of variance in change in weight accounted for. The relation of change in depression with waist circumference change was also significant (Table 2). Stepping in changes in self-efficacy to control eating when negative emotions are present, and self-regulation for eating, also significantly increased the portion of the variance accounted for. In both multiple regression equations, only change in self-regulation for eating significantly contributed uniquely to the explained variances (Table 2).

## 4. Discussion

Contrary to Dunn and colleagues [14], findings suggest that even a low-moderate volume of exercise (e.g., 2 days/week at a moderate duration and intensity), which is considerably less than the recommended public health volume of at least 150 minutes per week, is associated with a significant reduction in depression in severely obese women. Also, a dose-response effect was not found. Although biochemical theories were not directly tested and, thus, cannot be discounted, behavioral explanations consistent with social cognitive and self-efficacy theory were supported. Given the evidence that the public health dose of exercise is *very* difficult to maintain [17], this finding is favorable for those affected by emotional eating and challenged with maintaining exercise when attempting weight loss. 

The finding that the inverse relationship between depression and self-efficacy to control emotional eating was completely mediated by increased self-regulation skill use has practical implications. For example, if an exercise program is supported by instruction in self-regulatory skills, these skills may carry over to feelings of control over overeating—especially when negative emotions such as depression are a depleting factor. While laboratory research overwhelmingly suggests that when 2 tasks are sequentially attempted, one's self-regulation ability is diluted [33], more recent findings intimate that when self-regulatory skills are specifically taught, they become stronger and more resilient for the second task (such as with exercise and reduced calorie eating) [34]. Hence, the premise that the primary value of exercise program participation for weight loss for obese persons is through the associated psychological changes (rather than caloric expenditure) [8] was supported. 

The moderate (but significant) amount of the variance in changes in weight (12%) and waist circumference (25%) accounted for by changes in depression, self-efficacy, and self-regulation, suggests that there may be additional behavioral variables yet to be identified for incorporation into a stronger prediction model. It is suggested that researchers extend this investigation to uncover such factors, especially ones that may be modifiable. The present design of testing intervention effects on variables derived from behavioral theory, and then subsequently assessing associations of those changes with outcomes such as weight loss, is advocated for the future. It allows for ongoing improvements of theory and treatment to be expedited in both a scientific and practical manner. The addition of waist circumference change as an outcome measure was an advantage. It is suggested that outcome measures in addition to body weight be used in treatment research that incorporates exercise because associated increases in lean muscle may distort effects on weight [35].

Several limitations of this research should be noted, however. This study was conducted in a field setting, and thus subject to possible problems with internal validity. For example, social support and expectation effects may have biased responses. Practical settings have, however, been strongly advocated because of their ease in generalizing findings to practice [36]. Still, the sample of severely obese women tested does not allow generalization of findings to men, adolescents, or women of an unhealthy but lower weight. Although post hoc selection of groups by exercise volume completed may be viewed as problematic, in reality, adherence to a program of physical activity is highly varied [22]. As with previous weight-loss research [37], treatment results may be most generalizable when subject variations are accommodated within research designs [36]. Replication and extension of the present research, including with a control group, is needed to address the poor results consistently found in the array of weight-management treatments presently available [38].

##  Conflicts of Interest

The authors declare that there is no conflict of interest in the study.

## Figures and Tables

**Table 1:** Changes in measures over 24 weeks in an investigation on exercise and emotional eating with severely obese women, 2011 (*N* = 88).

Measure	Baseline Mean ± SD	Week 24 Mean ± SD	* t*(87)	*P*	95% CI	Change Mean ± SD	Skw^a^	Kurt^b^
Depression	4.09 ± 3.64	3.36 ± 3.67	−2.53	.01	−1.29, −0.17	−0.73 ± 2.65	−.71	1.94
Negative emotions	18.84 ± 10.05	22.13 ± 9.36	3.91	<.001	1.61, 4.95	3.28 ± 7.88	.75	2.17
Self-regulation for eating	22.13 ± 9.36	25.94 ± 6.81	5.60	<.001	2.35, 4.94	3.65 ± 6.11	.51	0.44
Weight (kg)	116.06 ± 17.88	113.63 ± 16.73	−5.49	<.001	−3.31, −1.55	−2.43 ± 4.16	—	—
Waist circum. (cm)	120.95 ± 13.69	117.87 ± 13.07	−4.70	<.001	−4.38, −1.78	−3.08 ± 6.14	—	—

Negative emotions = Weight Efficacy Lifestyle Scale-negative emotions subscale (self-efficacy to control eating when negative emotions are present).

^a^For skewness (Skw), SE =.26.

^b^For kurtosis (Kurt), SE =.51.

An empty cell (—) indicates that data does not apply.

**Table 2:** Results of multiple regression analyses predicting weight and waist circumference changes, 2011 (*N* = 88).

	*β*	SE	*R* ^2^	*P*	Δ*R* ^2^	*P*
Prediction of weight change						
Step 1			.04	.05		
Δ Depression	.21	.17		.05		
Step 2			.12	.01	.08	.03
Δ Depression	.05	.19		.68		
Δ Negative emotions	−.07	.07		.57		
Δ Self-regulation for eating	−.28	.09		.05		

Prediction of waist circumference change						
Step 1			.10	.002		
Δ Depression	.32	.24		.002		
Step 2			.25	.001	.15	.001
Δ Depression	.10	.25		.36		
Δ Negative emotions	−.03	.09		.78		
Δ Self-regulation for eating	−.42	.13		.001		

Negative emotions = Weight Efficacy Lifestyle Scale—negative emotions subscale (self-efficacy to control eating when negative emotions are present).

The delta symbol (Δ) denotes score change from baseline to week 24.
